# CO_2_‐Assisted Controllable Synthesis of PdNi Nanoalloys for Highly Selective Hydrogenation of Biomass‐Derived 5‐Hydroxymethylfurfural

**DOI:** 10.1002/anie.202418234

**Published:** 2024-11-19

**Authors:** Ruichao Guo, Yongjian Zeng, Lu Lin, Di Hu, Chunqiang Lu, Stuart Conroy, Suyu Zhang, Chen Zeng, Huixia Luo, Zhiwei Jiang, Xiaolei Zhang, Xin Tu, Kai Yan

**Affiliations:** ^1^ School of Environmental Science and Engineering Sun Yat-sen University Guangzhou 510275 China; ^2^ School of Environmental Science and Engineering Guangdong University of Technology Guangzhou 510006 China; ^3^ School of Materials Science and Engineering Sun Yat-sen University Guangzhou 510275 China; ^4^ Department of Electrical Engineering and Electronics University of Liverpool Liverpool L69 3GJ UK; ^5^ Department of Chemical and Process Engineering University of Strathclyde Glasgow G1 1XJ UK

**Keywords:** Biomass, 2,5-Bishydroxymethyltetrahydrofuran, Hydrogenation, 5-Hydroxymethylfurfural, Nanoalloys

## Abstract

The selective hydrogenation of 5‐hydroxymethylfurfural (HMF) to 2,5‐bishydroxymethyltetrahydrofuran (BHMTHF), a vital fuel precursor and solvent, is crucial for biomass refining. Herein, we report highly selective and stable PdNi nanoalloy catalysts for this deep hydrogenation process. A CO_2_‐assisted green method was developed for the controllable synthesis of various bimetallic and monometallic catalysts. The PdNi/SBA‐15 catalysts with various Pd/Ni ratios exhibited a volcano‐like trend between BHMTHF yield and Pd/Ni ratio. Among all catalysts tested, Pd_2_Ni_1_/SBA‐15 achieved the best performance, converting 99.0 % of HMF to BHMTHF with 96.0 % selectivity, surpassing previously reported catalysts. Additionally, the Pd_2_Ni_1_/SBA‐15 catalyst maintained excellent stability even after five recycling runs. Catalyst characterizations (e. g., HAADF‐STEM) and density functional theory (DFT) calculations confirmed the successful formation of the alloy structure with electron transfer between Ni and Pd, which accounts for the remarkable performance and stability of the catalyst. This work paves the way for developing highly selective and stable alloy catalysts for biomass valorization.

## Graphical abstract

A highly selective and stable Pd_2_Ni_1_/SBA‐15 nanoalloy catalyst has been prepared using a solvent‐free CO_2_‐assisted method for the hydrogenation of 5‐hydroxymethylfurfural. A striking volcano‐like trend was observed between activity and the Pd/Ni ratio, with 2,5‐bishydroxymethyltetrahydrofuran yield surpassing most previously reported catalysts. Alloying Pd with Ni and the formation of Pd^δ−^−Ni^δ+^ pairs significantly enhance catalytic performance.

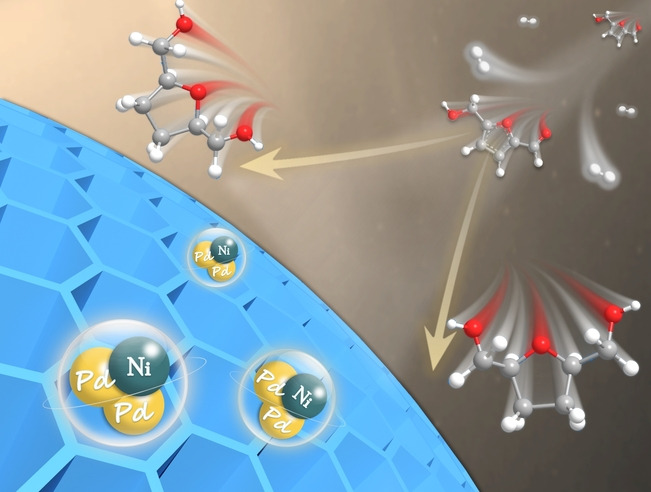

## Introduction

Biomass derivatives are attractive renewable alternatives for producing high‐value liquid fuels, fine chemicals, and polymer materials owing to their abundance, global availability, and carbon neutrality.[^1^, ^2^, ^3^] Among these promising biomass‐based platform molecules, 5‐hydroxymethylfurfural (HMF) stands out as a vital bridge linking biomass resources and value‐added chemicals.[^4^, ^5^, ^6^] The reactive functional groups (−CHO and −CH_2_OH) in HMF allow for a variety of reactions, including hydrogenation, hydrodeoxygenation, amination, oxidation, and other reactions, resulting in the production of 2,5‐bishydroxymethyltetrahydrofuran (BHMTHF), dimethylfuran (DMF), 2,5‐bis(aminomethyl)furan (BAMF), 2,5‐furandicarboxylic acid (FDCA), and others.[^7^, ^8^] Notably, BHMTHF is an environmentally friendly aprotic polar solvent with a high market value of around $10000 per ton. Moreover, BHMTHF can be used as a feedstock for producing 2,5‐dimethyltetrahydrofuran, a green biofuel. 1,6‐hexanediol (1,6‐HDO), a key monomer for producing polyurethanes, can also be prepared via the ring‐opening hydrogenolysis of BHMTHF.[^9^, ^10^] Therefore, the hydrogenation of HMF to BHMTHF holds great significance in sustainable biomass valorization.^[4]^

Converting HMF to BHMTHF requires selective hydrogenation of both the aldehyde group and the furan ring. Pd‐based catalysts, such as Pd/C, are widely used in hydrogenation processes[^11^, ^12^] due to their exceptional catalytic efficiency.[^13^, ^14^] However, Pd suffers from limitations such as a high energy barrier for ring hydrogenation and side reactions like hydrodeoxygenation and decarbonylation that reduce BHMTHF selectivity.^[4]^ Alloying Pd with another metal can effectively modulate its electronic and geometric structure due to mutual interaction, thus optimizing the adsorption of intermediates, leading to improved selectivity of the catalyst towards the target product.[^15^, ^16^] For instance, Tan et al.^[17]^ reported on the alloying effects in the hydrogenation of HMF to BHMTHF process. The reduced graphene oxide (RGO)‐supported bimetallic RuPd/RGO catalyst showed a higher yield of BHMTHF (92.9 %) compared to single metal Ru/RGO (6.0 %) and Pd/RGO (4.1 %) catalysts. Despite the excellent performance of RuPd/RGO in the HMF hydrogenation reaction, it is less practical for industrial production due to the use of dual noble metals with high cost and low availability. Non‐noble metals such as Ni,[^18^, ^19^] Co,[^20^, ^21^] and Cu,[^22^, ^23^] which exhibit efficient hydrogenation activity, are thus considered promising alternatives. Nakagawa et al.^[24]^ explored a SiO_2_‐supported Ni−Pd composite to facilitate the hydrogenation of HMF. The yield of BHMTHF could reach 96 %, demonstrating higher activity than the monometallic counterparts. However, the issue of Ni leaching remains prominent even after the first recycling run, leading to catalyst deactivation. Therefore, providing extremely efficient alloy catalysts with high stability for the selective hydrogenation of HMF remains challenging yet essential.

Supporting the active metal species on a porous material can effectively overcome leaching issues due to the steric effect.[^25^, ^26^, ^27^] Moreover, a suitable carrier can further enhance the alloying effect. Mesoporous silica materials, like SBA‐15, with their uniform pore sizes and high surface area, have gained significant interest as support materials for biomass conversion due to their ability to disperse catalysts effectively.[^28^, ^29^, ^30^] Traditional impregnation methods, commonly used to load nanoalloys onto mesoporous silica, often involve cumbersome washing and separation steps using hazardous solvents. A promising alternative is the CO_2_‐assisted impregnation method for fabricating nanoalloys. This method offers several advantages: solvent‐free synthesis, precise control over particle shape, size, and composition of metal particles, and overall green and efficient production of well‐defined nanoalloys.[^31^, ^32^] This makes CO_2_‐assisted impregnation a highly attractive strategy for developing catalysts for selective HMF hydrogenation.

Herein, we report the fabrication of robust and stable PdNi/SBA‐15 nanoalloy catalysts using a green CO_2_‐assisted synthesis method. These catalysts efficiently achieve deep hydrogenation of HMF to BHMTHF. We successfully synthesized a series of bimetallic PdNi/SBA‐15 with varying ratios, alongside monometallic Pd/Ni/Cu/Ru/SBA‐15 catalysts were successfully constructed. The physicochemical properties of the as‐obtained materials were comprehensively characterized using transmission electron microscopy (TEM), X‐ray diffraction (XRD), H_2_‐temperature programmed reduction (H_2_‐TPR), and X‐ray photoelectron spectroscopy (XPS). The as‐prepared bimetallic PdNi/SBA‐15 with different Pd/Ni ratios and monometallic Pd/Ni/Cu/Ru/SBA‐15 catalysts exhibited volcano‐like behavior in the deep hydrogenation of biomass‐derived HMF. Among them, the Pd_2_Ni_1_/SBA‐15 catalyst demonstrated the best performance, achieving 99.0 % conversion and 96.0 % selectivity for BHMTHF. This performance significantly surpasses most previously reported catalysts. Density functional theory (DFT) calculations revealed that the Pd^δ−^ and Ni^δ+^ sites formed on Pd_2_Ni_1_/SBA‐15 enhance the adsorption and activation of HMF, leading to high selectivity for BHMTHF. Notably, the Pd_2_Ni_1_/SBA‐15 nanoalloy catalysts maintained their stable structure and excellent catalytic activity even after five recycling runs.

## Results and Discussion

**Figure 1:**
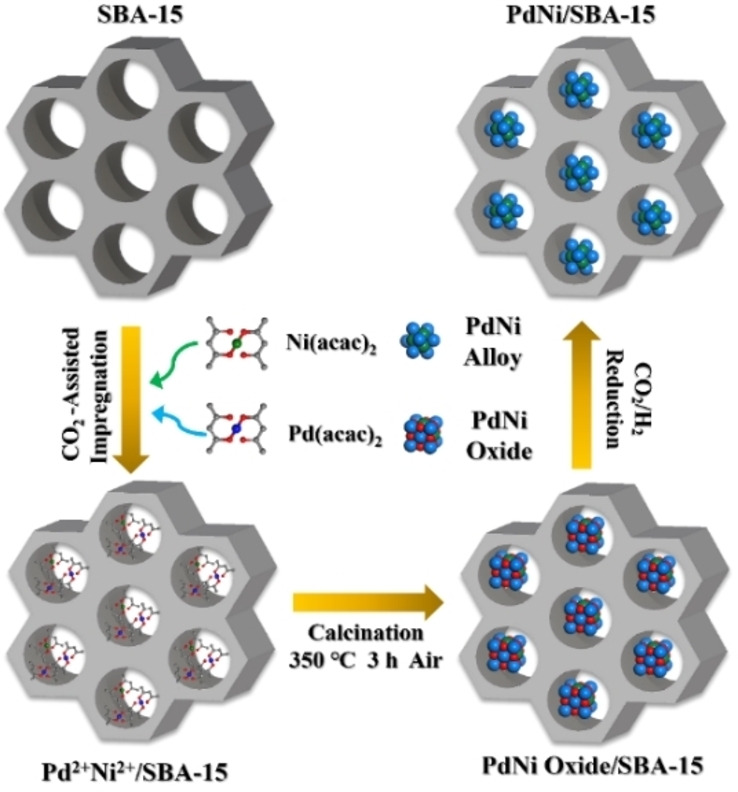
Synthesis process of PdNi alloy catalysts.

The PdNi bimetallic catalysts were synthesized employing a CO_2_‐assisted method (Figure 1). SBA‐15 was first prepared as the support material for loading the metal species. N_2_ physisorption analysis revealed that the obtained SBA‐15 exhibited an IV‐type isotherm (Figure S1), with a typical hysteresis loop of H1‐type and a pore size distribution centered around 6.1 nm, indicative of its mesoporous nature. This mesoporous structure is expected to facilitate the formation of well‐dispersed stable nanoalloy particles.^[33]^ The BET (Brunauer, Emmett and Teller) surface area of the SBA‐15 support was calculated to be 642 m^2^ g^−1^ and the Barrett‐Joyner‐Halenda (BJH) pore volume was 0.77 cm^3^ g^−1^.

Following CO_2_‐assisted impregnation, the desired metal precursors (palladium and nickel acetylacetonates in a specific molar ratio) were loaded onto SBA‐15 to achieve uniform dispersion of the metal species. To identify the optimal calcination temperature, thermogravimetric (TG) analysis was performed on the Pd^2+^Ni^2+^/SBA‐15 precursor (Figure S2). The TG results showed two distinct weight loss stages: 30–90 °C and 90–280 °C. These stages likely correspond to the desorption of physically adsorbed H_2_O and the decomposition of P123, respectively. Since the weight remained stable above 350 °C, this temperature was chosen for catalyst calcination. Finally, the PdNi alloy catalysts were obtained by reduction at 30 °C under a 30 vol% H_2_/CO_2_ atmosphere. These catalysts were named Pd_x_Ni_y_/SBA‐15, in which x and y refer to the mass ratio of Pd and Ni determined by inductively coupled plasma mass spectrometry (Table S1). Using the same CO_2_‐assisted method, a series of monometallic (Ni, Cu, Ru, and Pd) and bimetallic (Pd_1_Ru_1_, Pd_1_Cu_1_, Pd_1_Ni_1_, Pd_1_Ni_2_, and Pd_2_Ni_1_) catalysts were synthesized (Figure S3).

**Figure 2:**
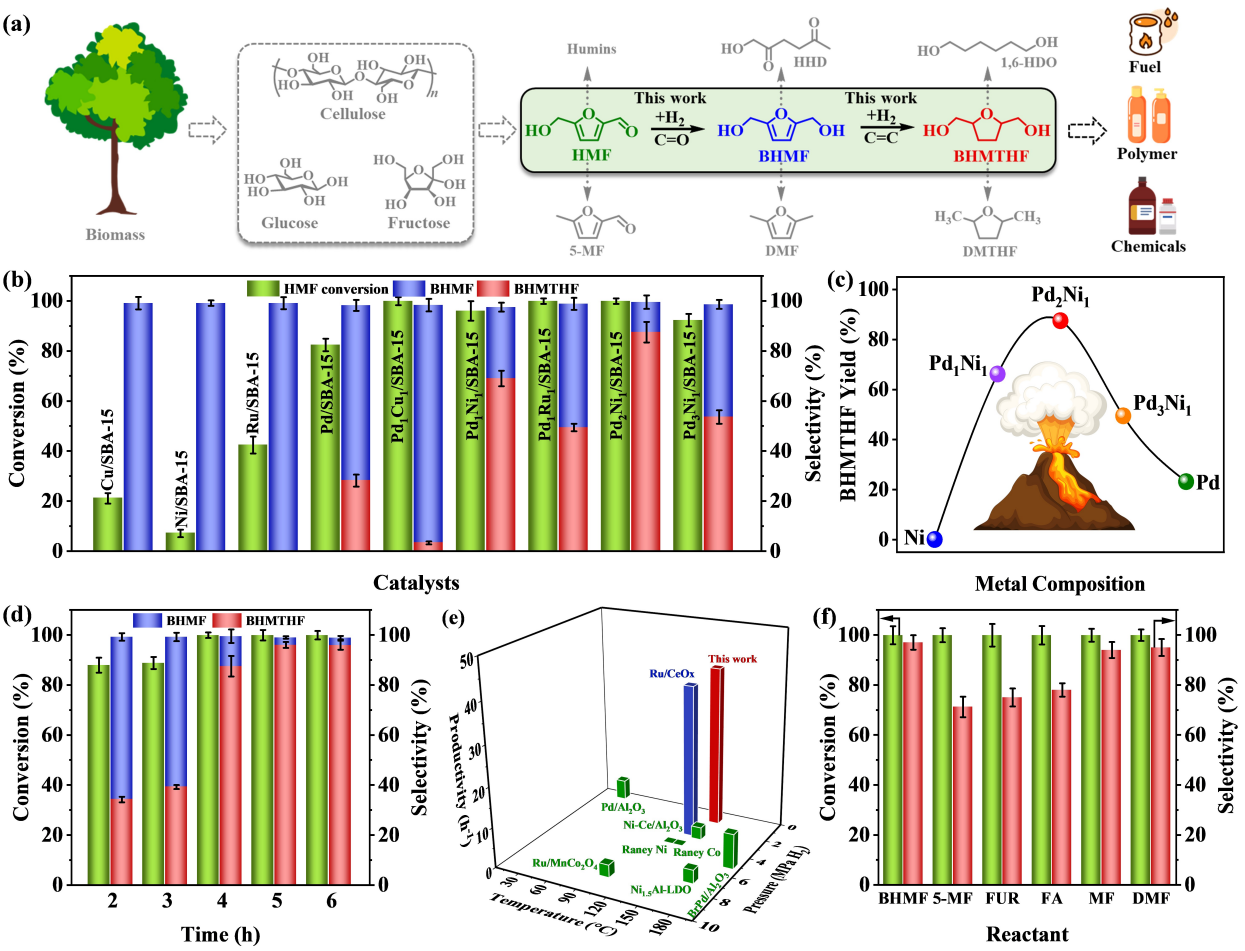
(a) Production route of BHMTHF from lignocellulosic biomass; (b–c) Catalytic performance of different catalysts for HMF hydrogenation to BHMTHF (140 °C, 4 h, 1 MPa H_2_); (d) Effect of reaction time (140 °C, 1–6 h, 1 MPa H_2_) on the catalytic performance of Pd_2_Ni_1_/SBA‐15 for HMF hydrogenation to BHMTHF; (e) Comparison with other catalysts for HMF hydrogenation to BHMTHF; (f) Hydrogenation reaction of BHMF (140 °C, 5 h, 1 MPa H_2_), 5‐methyl furfural (5‐MF: 140 °C, 12 h, 3 MPa H_2_), furfural (FUR: 140 °C, 12 h, 3 MPa H_2_), furfural alcohol (FA: 140 °C, 12 h, 3 MPa H_2_), 2‐methylfuran (MF: 150 °C, 12 h, 2 MPa H_2_), and 2,5‐dimethylfuran (DMF: 150 °C, 12 h, 2 MPa H_2_) over Pd_2_Ni_1_/SBA‐15.

The hydrogenation of HMF to BHMTHF is a crucial reaction in biomass refineries as it enables the conversion of biomass‐based compounds into valuable chemicals and fuels.[^9^, ^34^, ^35^] HMF is typically produced from cellulose hydrolysis followed by isomerization and dehydration. BHMTHF generated from HMF hydrogenation finds numerous applications in the production of fuel additives, polymers, and various chemicals (Figure 2a). The HMF hydrogenation reaction was conducted in a batch reactor (Figure S4) at 140 °C and 1 MPa H_2_ for four hours, using 1,4‐dioxane as the solvent. As illustrated in Figure 2b, the Pd/SBA‐15 monometallic catalyst exhibited 82.4 % HMF conversion of HMF but achieved only 28.2 % selectivity for BHMTHF. Notably, Cu/SBA‐15, Ni/SBA‐15, and Ru/SBA‐15 catalysts demonstrated lower activity for HMF hydrogenation, resulting in limited BHMTHF yields. The bimetallic catalysts (Pd_1_Cu_1_/SBA‐15, Pd_1_Ni_1_/SBA‐15, and Pd_1_Ru_1_/SBA‐15) catalysts prepared through alloying with Pd showed significantly enhanced activity than the monometallic counterparts, which is likely benefit from the synergistic effects between Pd and the other metals. Among the tested catalysts, Pd_1_Ni_1_/SBA‐15 demonstrated the best performance, achieving 96.0 % conversion of HMF and 69.0 % selectivity of BHMTHF. The turnover frequency (TOF) value and initial specific activity of Pd_2_Ni_1_/SBA‐15 were much higher than those of Pd/SBA‐15, indicating the superior catalytic activity of Pd_2_Ni_1_/SBA‐15 (Table S2). Furthermore, the influence of the metal ratio in PdNi/SBA‐15 on their activity for HMF hydrogenation to BHMTHF was investigated. As shown in Figure 2c, a volcano‐like trend was observed. The Pd_2_Ni_1_/SBA‐15 catalyst displayed a higher BHMTHF yield (87.5 %) compared to Pd_1_Ni_1_/SBA‐15 (69.0 %) and Pd_1_Ni_2_/SBA‐15 (55.2 %), indicating that an optimal Pd to Ni ratio has a crucial impact on optimizing the activity of the bimetallic catalyst by promoting the alloying effects between Pd and Ni.

To further optimize the BHMTHF yield, key reaction parameters were investigated. First, the hydrogenation reaction was conducted at various temperatures (1 MPa H_2_ and 4 h) to examine the temperature dependence (Figure S5a). As the temperature increased from 80 °C to 140 °C, both HMF conversion and BHMTHF selectivity improved significantly, rising from 80.3 % to 99.9 % and 25.2 % to 87.5 %, respectively. However, further increasing the temperature to 160 °C resulted in a substantial decrease in BHMTHF selectivity (22.4 %). This is likely due to the cleavage of C−OH bonds at higher temperatures, as evidenced by the formation of 2,5‐dimethyl tetrahydrofuran (DMTHF) as a by‐product. Therefore, the optimal reaction temperature was 140 °C. The effects of metal loading and solvent on the conversion of HMF and the selectivity of BHMTHF were also investigated, with the results shown in Figure S5b–c. Using 1,4‐dioxane as the solvent and a metal loading of 2 wt% is optimal for the conversion of HMF over Pd_2_Ni_1_/SBA‐15 catalyst. The reaction time was also explored to assess its impact on catalytic performance (Figure 2d). Prolonging the time from 2 h to 5 h led to an increase in both HMF conversion (87.9 % to 99.9 %) and BHMTHF selectivity (34.2 % to 96.0 %). However, no significant changes were observed after extending the reaction time to 6 h. Subsequently, the influence of H_2_ pressure on the catalytic activity of Pd_2_Ni_1_ was further investigated (Figure S5d). The results indicate that 5 h and 1 MPa H_2_ are sufficient to achieve an optimized BHMTHF yield of 96.0 %, corresponding to a high productivity of 40 h^−1^, which is three times higher than the Pd/SBA‐15 catalyst. This performance surpasses most previously reported catalysts (Figure 2e, Table S3), which afforded high activity at long reaction time (>10 h) and high H_2_ pressure (>3 MPa).[^36^, ^37^, ^38^, ^39^, ^40^, ^41^, ^42^] These results demonstrate the exceptional performance of the Pd_2_Ni_1_/SBA‐15 alloy catalyst for HMF hydrogenation to BHMTHF. The universality of Pd_2_Ni_1_/SBA‐15 was then evaluated by expanding the reactant to BHMF, 5‐methyl furfural (5‐MF), furfural (FUR), furfural alcohol (FA), 2‐methylfuran (MF), and 2,5‐dimethylfuran (DMF) (Figure 2f). All the substrates were fully converted within the desired time. The high yield of BHMTHF, 5‐methyltetrahydrofurfuryl alcohol, tetrahydrofurfuryl alcohol, 2‐methyltetrahydrofuran, and 2,5‐dimethyltetrahydrofuran were obtained from BHMF, 5‐MF, FUR, FA, MF and DMF, respectively, indicating Pd_2_Ni_1_/SBA‐15 as an efficient catalyst for the hydrogenation of furan ring.

**Figure 3:**
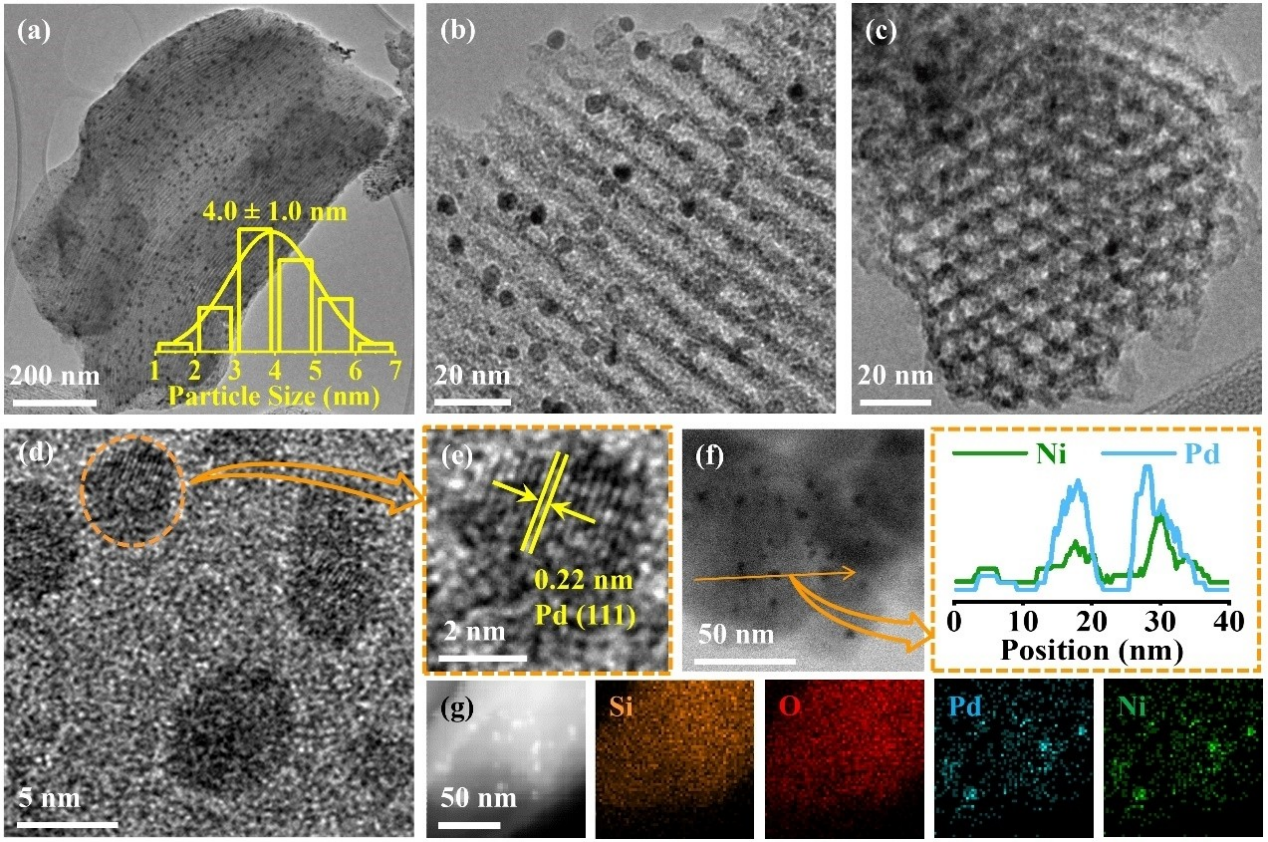
(a) TEM images, (b–e) HR‐TEM images, (f) line profiles, (g) HAADF‐STEM image, and EDX‐elemental mapping results of Pd_2_Ni_1_/SBA‐15 catalyst.

Since Pd_2_Ni_1_/SBA‐15 exhibited the highest activity among the investigated catalysts, its physicochemical properties were thoroughly analyzed. The TEM image in Figure 3a confirms the ordered mesoporous structure of the Pd_2_Ni_1_/SBA‐15 catalyst. Metal particles are homogeneously dispersed throughout the catalyst with an average diameter of 4.0±1.0 nm, as determined from analysis of over 100 bimetallic particles (Figure S6). TEM images from various perspectives (Figure 3b–c) further reveal that the PdNi nanoalloys are primarily located within the channels of the SBA‐15 support. High‐resolution transmission electron microscopy (HR‐TEM) images (Figure 3d–e) show that the Pd_2_Ni_1_/SBA‐15 catalyst consists of nanoparticles (NPs) corresponding to the PdNi crystal structure. The measured d‐spacing of 0.22 nm aligns well with the (111) plane of the PdNi lattice (0.217 nm). The energy dispersive spectroscopy (EDS) line scan of metal NPs (Figure 3f and Figure S7a–c) confirms the uniform dispersion of Ni and Pd, and the formation of a PdNi nanoalloy. Additionally, the element mappings (Figure 3g and Figure S7d) for Pd and Ni further corroborate the presence of PdNi nanoalloys within the Pd_2_Ni_1_/SBA‐15 catalyst.

**Figure 4:**
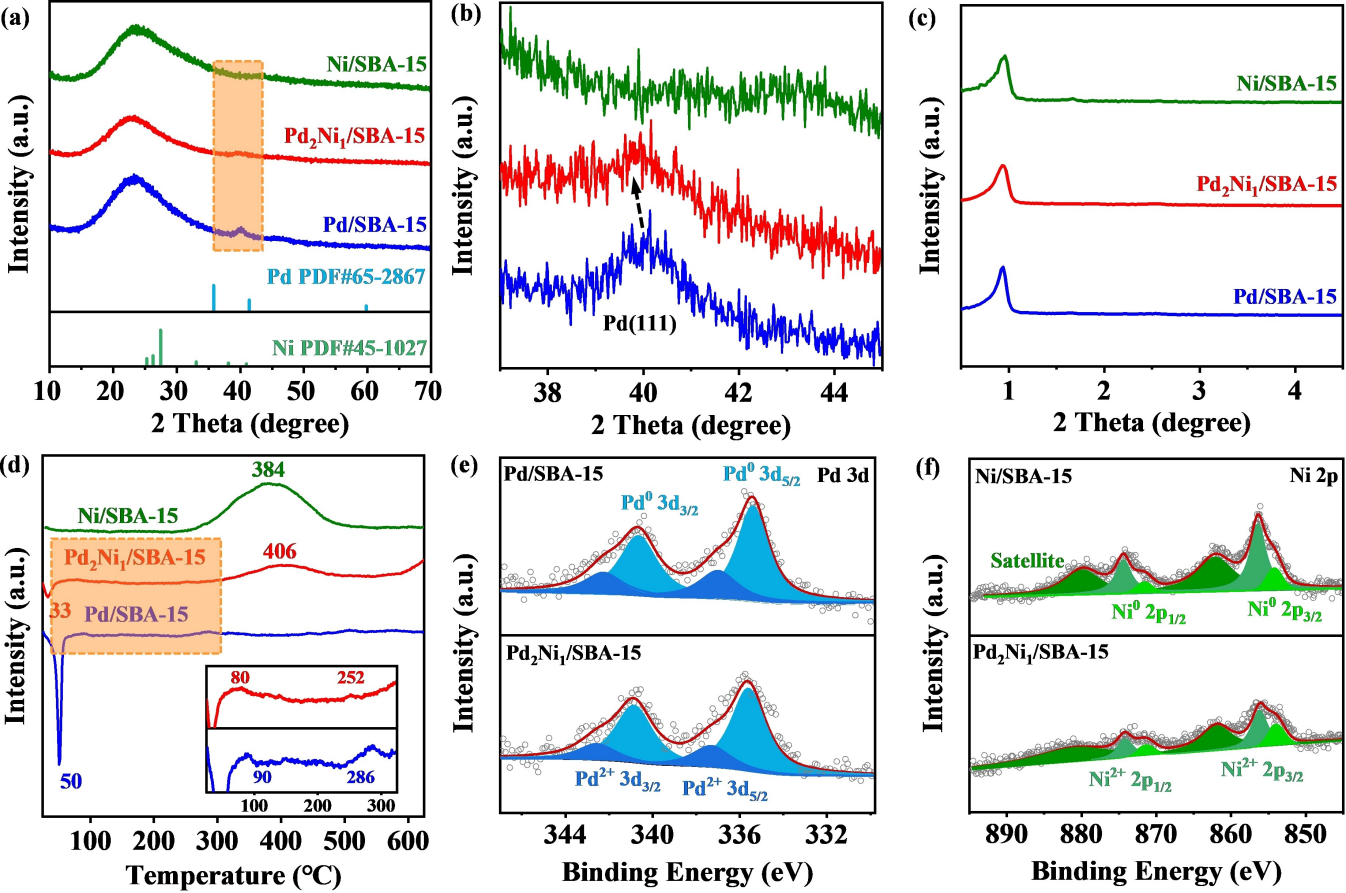
(a) XRD patterns, (b) zoomed‐in XRD patterns between 36.0° and 46.0°, and (c) low‐angle XRD patterns of Ni/SBA‐15, Pd_2_Ni_1_/SBA‐15, and Pd/SBA‐15; (d) H_2_‐TPR profiles of Ni/SBA‐15, Pd_2_Ni_1_/SBA‐15, and Pd/SBA‐15; (e) Pd 3d XPS spectra of Pd/SBA‐15 and Pd_2_Ni_1_/SBA‐15; (f) Ni 2p XPS spectra of Ni/SBA‐15 and Pd_2_Ni_1_/SBA‐15.

To elucidate the improved catalytic performance of the Pd_2_Ni_1_/SBA‐15 catalyst for HMF hydrogenation to BHMTHF, its physiochemical properties were compared to those of the monometallic Pd/SBA‐15 and Ni/SBA‐15 catalysts. Scanning electron microscope (SEM) images revealed similar morphologies for all three catalysts (Figure S8–S10). TEM images (Figure 3, Figure S11) and scanning transmission electron microscopy (STEM) images (Figure S9c) of the different catalysts confirmed that the average sizes of nanoparticles in the series of catalysts are similar. The XRD patterns are displayed in Figure 4a. The broad peak centered at ~24° originates primarily from the amorphous SBA‐15 support. For Pd_2_Ni_1_/SBA‐15 and Pd/SBA‐15, weak peaks can be observed around 40°, corresponding to the Pd (111) plane (JCPDS card no. 65‐2867).[^43^, ^44^] The average size of PdNi NPs, estimated using the Scherrer equation, was around 4 nm for Pd_2_Ni_1_/SBA‐15, which aligns well with the TEM analysis. Interestingly, a closer look at the 2θ range of 37.0°–45.0° (Figure 4b) reveals a slight shift in the Pd diffraction peak of the Pd_2_Ni_1_/SBA‐15 catalyst compared to Pd/SBA‐15. This shift towards a lower angle suggests the incorporation of smaller Ni atoms into the Pd lattice, resulting in the formation of alloy phases.^[45]^ However, distinct peaks for Ni (JCPDS card no. 45‐1027) were barely detectable in the Pd_2_Ni_1_/SBA‐15 and Ni/SBA‐15 catalysts. This might be attributed to the low Ni loading and the strong driving force for crystallization during synthesis.^[46]^ The low‐angle XRD patterns (Figure 4c) exhibit an obvious peak at around 0.97°, primarily arising from the mesopores of the SBA‐15 support.

H_2_−TPR experiments were then conducted on the three representative catalysts to investigate their reduction properties, as shown in Figure 4d. For both Pd_2_Ni_1_/SBA‐15 and Pd/SBA‐15 catalysts, a negative peak is observed at ~33 °C and ~50 °C, respectively. This peak corresponds to the decomposition of Pd hydride (β−PdH_x_) formed during H_2_ flushing at low temperatures. It is reported that larger particles can absorb more hydrogen molecules compared to smaller ones, resulting in a higher peak intensity.[^47^, ^48^] Therefore, the significantly weaker negative peak intensity observed for Pd_2_Ni_1_/SBA‐15 compared to Pd/SBA‐15 suggests the formation of smaller Pd nanoparticles after alloying with Ni. The two broad peaks observed in the temperature range of 25–325 °C derive from the reduction of surface PdO and more strongly bound cationic Pd species interacting with the support.^[46]^ Moreover, the H_2_ consumption signal associated with the reduction of Ni oxides in Pd_2_Ni_1_/SBA‐15 (406 °C) appears at a higher temperature compared to Ni/SBA‐15 (384 °C). This finding further corroborates the existence of an interaction between Pd and Ni in the Pd_2_Ni_1_/SBA‐15 catalyst.

XPS analysis was performed to compare the surface chemical states of Ni and Pd on Ni/SBA‐15, Pd/SBA‐15, and Pd_2_Ni_1_/SBA‐15 catalysts (Figure S12). The Pd 3d spectrum of Pd_2_Ni_1_/SBA‐15 (Figure 4e and Table S4) exhibits two main peaks corresponding to Pd^0^ (metallic palladium) at binding energies of 335.6 eV and 340.8 eV, along with two smaller peaks for Pd^2+^ at 337.3 eV and 342.5 eV.^[49]^ In the Ni 2p region (Figure 4f and Table S4), the peaks at 854.0 and 871.4 eV represent Ni^0^ species. The broader peaks at 856.2/874.6 eV and their corresponding satellite signals at 861.8/881.2 eV can be ascribed to the existence of Ni oxides.[^50^, ^51^]

**Figure 5:**
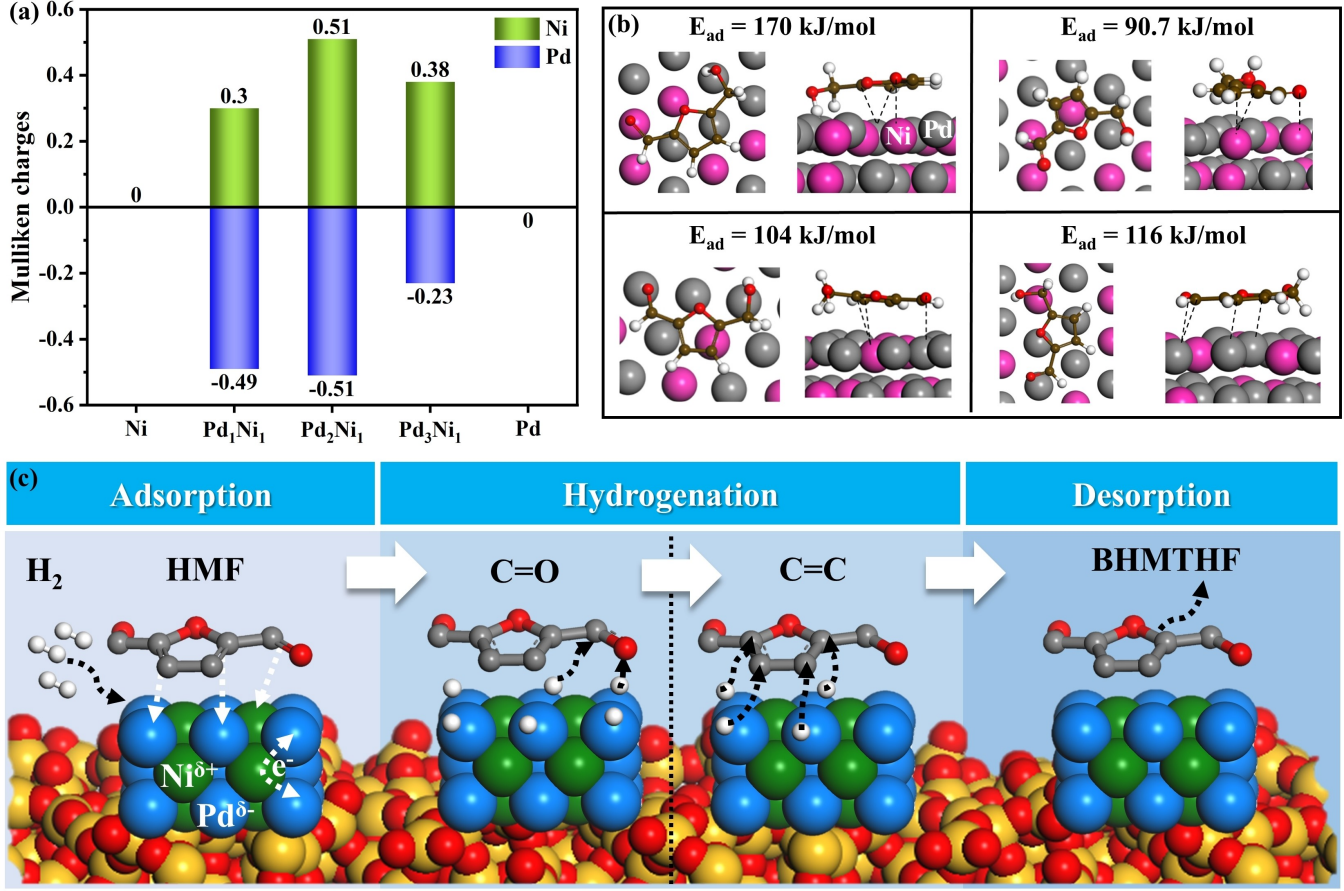
(a) Atomic population analysis based on the Mulliken charges of Ni and Pd atoms in various catalyst alloys. Positive values correspond to a loss of electrons, while negative values represent a gain of electrons; (b) Various adsorption configurations for HMF on Pd_2_Ni_1_ catalyst surface; (c) Proposed mechanisms of HMF hydrogenation to BHMTHF over Pd_2_Ni_1_/SBA‐15.

Additionally, atomic population analysis was carried out to analyze further the electron transfer behavior between Pd and Ni atoms within PdNi alloy catalysts. The Mulliken charges of Ni and Pd in various catalysts (Figure 5a) suggest that electrons are transferred from Ni to Pd, leading to the formation of electron‐enriched Pd^δ−^ (negative Mulliken charges) species and electron‐deficient Ni^δ+^ (positive Mulliken charges) in the PdNi/SBA‐15 catalysts. This conclusion that electron transfer from Ni to Pd in PdNi alloy catalysts is consistent with the electronegativities of Ni and Pd.[^52^, ^53^, ^54^] This electronic interplay between Pd and Ni in the Pd_2_Ni_1_/SBA‐15 catalyst likely enhances the adsorption and activation of HMF molecules.[^55^, ^56^] This improved activation is believed to be responsible for the significantly higher BHMTHF yield observed with the PdNi bimetallic sites compared to the monometallic catalysts. More importantly, the atomic populations analysis results shown in Figure 5a further confirm that the optimal synergistic effect occurs when the ratio of Pd to Ni is 2 : 1. This effect gradually diminishes with increasing proportions of either Pd or Ni, leading to a volcano‐like trend between the yield of BHMTHF and the metal composition of the catalyst.

DFT calculations were conducted in depth to investigate the reaction mechanism for converting HMF into BHMTHF. The hydrogenation activation energies of the unsaturated C=C and C=O bonds in HMF were calculated. The activation energy for the hydrogenation of the C=O bond (250 kJ/mol) is lower than that of the C=C bond (300 kJ/mol), indicating that the preferred reaction pathway for converting HMF to BHMTHF is through the hydrogenation of the aldehyde group, followed by the hydrogenation of furan‐ring. To further elucidate the HMF hydrogenation on the catalysts, the adsorption of H_2_ and HMF on Pd(111), Ni(111), and Pd_2_Ni_1_(111) were investigated, with the results shown in Figure S13. The highest adsorption energies of H_2_ (104 kJ/mol) and HMF (170 kJ/mol) on Pd_2_Ni_1_(111) suggest that H_2_ and HMF adsorb most easily on Pd_2_Ni_1_ alloy catalyst compared to the monometallic catalysts. Based on the electronic structure of Pd_2_Ni_1_ alloy catalyst, the adsorption structure of HMF on Pd_2_Ni_1_(111) surface was further analyzed by DFT. As shown in Figure 5b, the most stable adsorption configuration involves the interaction of Pd^δ−^ sites with furan‐ring and Ni^δ+^ sites with aldehyde group. Thus, the DFT results reveal the enhanced adsorption of H_2_ and HMF on Pd_2_Ni_1_ alloy catalyst, with the formed Pd^δ−^ and Ni^δ+^ species showing strong interaction with furan‐ring and aldehyde group, respectively. This leads to the high selectivity for BHMTHF over the Pd_2_Ni_1_ alloy catalyst. These results align well with the experimental findings.

Based on the preceding analysis, a possible mechanism for the enhanced BHMTHF yield using the Pd_2_Ni_1_/SBA‐15 bimetallic catalyst is proposed in Figure 5c. The first stage involves the adsorption and dissociation of H_2_ at the Pd active sites. The PdNi alloy structure in Pd_2_Ni_1_/SBA‐15, as confirmed by various characterization techniques (XRD, TEM, H_2_−TPR, XPS) and DFT calculations, is likely to facilitate H_2_ adsorption and activation during this step compared to Pd/SBA‐15. Simultaneously, HMF molecules adsorb onto the Pd_2_Ni_1_ nanoalloy surface. Notably, electron transfer occurs from Ni to Pd within the alloy, creating electron‐enriched Pd^δ−^ and electron‐deficient Ni^δ+^ sites. The Pd^δ−^ and Ni^δ+^ sites interact strongly with the C=O and C=C bonds of the HMF, respectively, exhibiting pronounced synergistic adsorption on the HMF molecule. This interaction facilitates the efficient activation of HMF, thereby promoting its deep hydrogenation. Consequently, the aldehyde group of the HMF molecules can efficiently bind with two hydrogen atoms, forming BHMF. The subsequent hydrogenation of the BHMF furan ring progresses smoothly, leading to the formation of the desired product, BHMTHF. Finally, BHMTHF desorbs from the surface of the PdNi nanoalloy, freeing up active sites for the next catalytic cycle.

**Figure 6:**
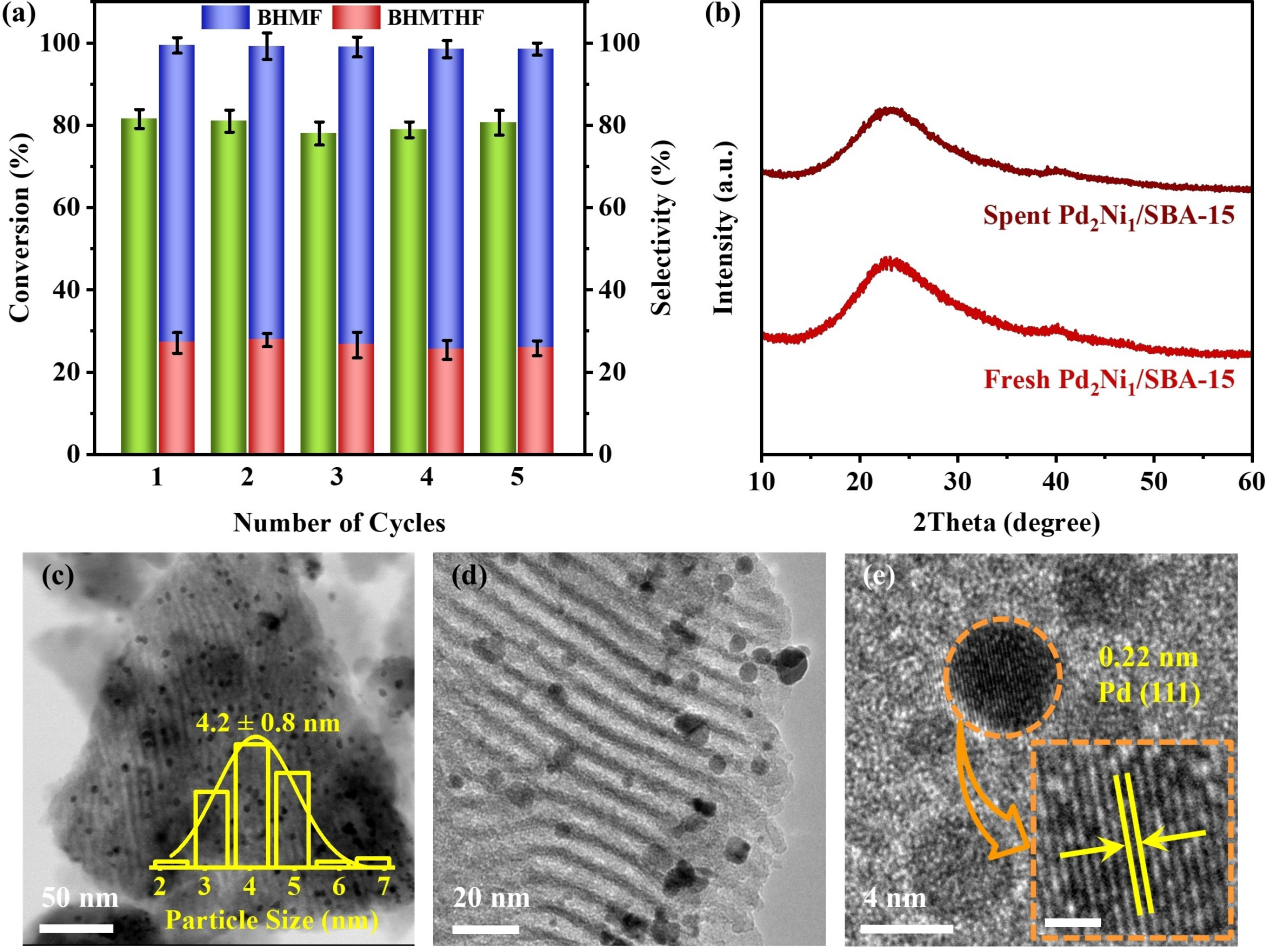
(a) Recycling performance of Pd_2_Ni_1_/SBA‐15 catalyst (80 °C, 4 h, 1 MPa H_2_); (b) XRD patterns of fresh and spent Pd_2_Ni_1_/SBA‐15 after the 5^th^ recycling cycle; (c–d) TEM images and (e) HR‐TEM image of spent Pd_2_Ni_1_/SBA‐15 catalyst after the 5^th^ recycling cycle.

The stability of the Pd_2_Ni_1_/SBA‐15 catalyst was evaluated through a recycling experiment. After each hydrogenation reaction, the spent catalyst was recovered using a separation and drying process before being subjected to a new reaction cycle. The results in Figure 6a and Figure S14 demonstrate that the Pd_2_Ni_1_/SBA‐15 catalyst can be reused for at least 5 consecutive cycles without a significant decrease in BHMTHF selectivity at both ~80 % and 100 % conversion of HMF. In addition, no substantial loss of metal content was observed in the spent catalyst after five cycles (Table S1). Inductively coupled plasma‐optical emission spectroscopy (ICP‐OES) testing of the solution after the reaction also indicated the absence of Ni and Pd species. The structural integrity of the Pd_2_Ni_1_/SBA‐15 catalyst after the recycling test was investigated using XRD and TEM analysis (Figure 6b–e and Figure S15). The results indicate that the Pd_2_Ni_1_/SBA‐15 catalyst maintains its crystallinity and the size of the nanoalloy particles remains relatively unchanged. Furthermore, XPS analysis of the spent catalyst (Figure S16) reveals that the chemical states of the metal components are well‐preserved. These combined observations provide strong evidence for the excellent stability of the Pd_2_Ni_1_/SBA‐15 catalyst during HMF hydrogenation to BHMTHF. The exceptional stability can likely be attributed to the alloying of Ni with Pd and the mesoporous structure of the SBA‐15 support. This combination may prevent metal leaching and aggregation during HMF hydrogenation, leading to an ultra‐stable Pd_2_Ni_1_/SBA‐15 catalyst.

## Conclusions

This study successfully synthesized bimetallic PdNi/SBA‐15 nanoalloy catalysts for the selective hydrogenation of HMF to BHMTHF. Notably, the Pd_2_Ni_1_/SBA‐15 catalyst exhibited exceptional performance, achieving nearly 100 % conversion of HMF and a remarkable 96.0 % selectivity toward BHMTHF under reaction conditions of 140 °C, 5 h, and 1 MPa H_2_. This performance surpasses that of monometallic (Ru, Pd, Cu, Ni) and other bimetallic (PdRu, PdCu, Pd_1_Ni_1_, Pd_3_Ni_1_) catalysts supported on SBA‐15 and is also outstanding compared to previously reported similar catalytic systems. The characterization results confirmed the formation of PdNi nanoalloys within the Pd_2_Ni_1_/SBA‐15 catalyst. The electronic transfer from Ni to Pd generates Pd^δ−^−Ni^δ+^ pairs, which are believed to be responsible for the efficient activation of not only the C=O group in the aldehyde but also the C=C bond in the furan ring of HMF, ultimately leading to the high BHMTHF yield. Expanding the reactant to BHMF and other biomass‐derived derivatives affords a high yield of the corresponding products over the Pd_2_Ni_1_/SBA‐15 nanoalloy. Additionally, alloying Pd with Ni mitigates metal leaching and aggregation during the catalytic process, contributing to the excellent stability of the Pd_2_Ni_1_/SBA‐15 catalyst. This work demonstrates a successful example of designing and fabricating highly selective and stable nanoalloy catalysts for the efficient upgrading of biomass resources into value‐added chemicals. By leveraging the synergistic effects of metal alloying and controlled synthesis methods, this approach paves the way for the development of advanced catalysts for biomass valorization.

## Supporting Information

The authors have cited additional references within the Supporting Information.

## Conflict of Interests

The authors declare no conflict of interest.

1

## Data Availability

The data that support the findings of this study are available from the corresponding author upon reasonable request.
